# An FGFR/AKT/SOX2 Signaling Axis Controls Pancreatic Cancer Stemness

**DOI:** 10.3389/fcell.2020.00287

**Published:** 2020-05-07

**Authors:** Mei-Yu Quan, Qiang Guo, Jiayu Liu, Ruo Yang, Jing Bai, Wei Wang, Yaxin Cai, Rui Han, Yu-Qing Lv, Li Ding, Daniel D. Billadeau, Zhenkun Lou, Saverio Bellusci, Xiaokun Li, Jin-San Zhang

**Affiliations:** ^1^School of Pharmaceutical Sciences and International Collaborative Center on Growth Factor Research, Wenzhou Medical University, Wenzhou, China; ^2^Institute of Life Sciences, Wenzhou University, Wenzhou, China; ^3^Center for Precision Medicine, The First Affiliated Hospital of Wenzhou Medical University, Wenzhou, China; ^4^Division of Oncology Research and Schulze Center for Novel Therapeutics, Mayo Clinic, Rochester, MN, United States; ^5^Cardio-Pulmonary Institute, Member of the German Lung Center, Justus Liebig University Giessen, Giessen, Germany

**Keywords:** FGFR, SOX2, pancreatic cancer, stemness, sphere-formation assay

## Abstract

Cancer stemness is associated with high malignancy and low differentiation, as well as therapeutic resistance of tumors including pancreatic ductal adenocarcinoma (PDAC). Fibroblast growth factors (FGFs) exert pleiotropic effects on a variety of cellular processes and functions including embryonic stem cell pluripotency and cancer cell stemness via the activation of four tyrosine kinase FGF receptors (FGFRs). FGF ligands have been a major component of the cocktail of growth factors contained in the cancer stemness-inducing (CSI) and organoid culture medium. Although FGF/FGFR signaling has been hypothesized to maintain cancer stemness, its function in this process is still unclear. We report that inhibition of FGF/FGFR signaling impairs sphere-forming ability of PDAC *in vitro*, and knocking down *FGFR1* and *FGFR2* decreased their tumorigenesis abilities *in vivo*. Mechanistically, we demonstrated that SOX2 is down-regulated upon loss of FGFR signaling. The overexpression of *SOX2* in SOX2-negative cells, which normally do not display stemness capabilities, is sufficient to induce spheroid formation. Additionally, we found that AKT phosphorylation was reduced upon FGFR signaling inhibition. The inhibition of AKT using specific pharmacological inhibitors in the context of CSI medium leads to the loss of spheroid formation associated with loss of SOX2 nuclear expression and increased degradation. We demonstrate that an FGFR/AKT/SOX2 axis controls cancer stemness in PDAC and therefore may represent an important therapeutic target in the fight against this very aggressive form of cancer.

## Introduction

Pancreatic ductal adenocarcinoma (PDAC) is a devastating disease because of its late diagnosis and resistance to various therapies. The overall survival rate of pancreatic cancer remains woefully low. Only a modest improvement in overall survival was achieved with the preferred regimens of adjuvant therapies such as FOLFIRINOX or gemcitabine combined with nab-paclitaxel (Conroy et al., 2011; Von Hoff et al., 2013; Oba et al., 2020).

*KRAS* oncogenic mutation is considered the most frequent and initial genetic event observed in approximately 90% of all PDAC. Activation of KRAS is a key element in the MAPK pathway, which is responsible for cell proliferation and survival. Most PDAC carrying oncogenic *KRAS* present deregulated cell growth and high mortality (Bryant et al., 2014). KRAS itself is difficult to inhibit and the effectiveness of agents that target key KRAS effectors failed therapeutically likely due to compensatory mechanisms (Manchado et al., 2016; Waters and Der, 2018). Several studies have demonstrated that multiple receptor tyrosine kinases (RTKs) including FGFRs display aberrant expression in PDAC (Motoda et al., 2011; Ishiwata et al., 2012; Lehnen et al., 2013), which is involved in regulating pancreatic acinar-to-ductal metaplasia (Shi et al., 2018). PDAC showed higher malignancy when treated with FGFs (Coleman et al., 2014). To date, inhibitors targeting FGFRs are useful adjuvants for PDAC therapy (Matsuda et al., 2014; Lai et al., 2018), suggesting that FGFRs display KRAS independent activities in enhancing cancer malignancy in PDAC.

FGF/FGFR is an important signal during mouse organogenesis (Teven et al., 2014; Ornitz and Itoh, 2015; Ndlovu et al., 2018), tissue repair/regeneration (Maddaluno et al., 2017; Tan et al., 2017, 2018). In humans, deregulation of the FGF/FGFR axis is involved in oncogenesis, tumor progression and resistance to anti-cancer treatment across multiple types of tumors (Dienstmann et al., 2014; Dianat-Moghadam and Teimoori-Toolabi, 2019). The FGFR family consists of four highly conserved transmembrane RTKs (FGFR1–4) and their aberrant activation gives rise to the activation of many cancer-related pathways, such as MAPK, PLCγ, PI3K/AKT, JAK/STAT (Ornitz and Itoh, 2015; Touat et al., 2015). This ultimately accelerates malignancy in cancer (Babina and Turner, 2017; Dianat-Moghadam and Teimoori-Toolabi, 2019), including stemness maintenance, proliferation, epithelial to mesenchymal transition (EMT), angiogenesis, etc. Cancer cells treated with FGFR inhibitors display, in many instances, an increased sensitivity to anti-cancer drugs (Katoh and Nakagama, 2014; Facchinetti et al., 2020). Additionally, FGF appears to be an indispensable supplementary growth factor in the cancer stemness-inducing (CSI) medium, and FGF2 in particular has been widely used to trigger spheroid formation *in vitro*. Despite the strong evidence suggesting that FGF/FGFR signal is indeed an essential factor in governing cancer stemness and a potential target for cancer therapy (Touat et al., 2015; Hallinan et al., 2016; Mossahebi-Mohammadi et al., 2020), the underlying mechanisms of how FGF/FGFR regulates cancer stemness are still unknown.

Cancer stem cells (CSCs) have been identified in many solid tumors, including skin, pancreas, brain and ovarian (Bjerkvig et al., 2005; Jordan et al., 2006; Li et al., 2007; Trumpp and Wiestler, 2008). The CSCs are related to tumor initiation, development, metastasis and recurrence of cancer (Pardal et al., 2003; Clevers, 2011). The concepts of PDAC stem cells in tumor initiation, resistance to therapeutic modalities, distant metastasis and cancer recurrence are increasingly documented (Li et al., 2013). PDAC cells enriched with CD44, and/or CD24 and/or CD133 (Hermann et al., 2007; Li et al., 2007; Hong et al., 2009; Ding et al., 2012; Salaria et al., 2015) are highly tumorigenic-resistant to conventional anti-cancer therapy, and have been used as cancer stemness markers (Ercan et al., 2017).

Sex-determining region Y (SRY)-Box2 (SOX2) protein, as a transcription factor, is responsible for coordinating disparate functions and maintaining stem cell properties as well as differentiation restriction (Sekido and Lovell-Badge, 2009; Wegner, 2010). For most cancers including PDAC, SOX2 expression has also been detected at the protein level by immunohistochemistry (Sanada et al., 2006; Laga et al., 2011). It is important to note that the corresponding normal pancreas, as well as the associated pre-malignant and pancreatic intraepithelial neoplasia, barely express SOX2. Poorly differentiated and modestly invasive tumors are associated in particular with increased expression of SOX2 protein (Sanada et al., 2006; Herreros-Villanueva et al., 2013). Importantly, down-regulation in SOX2 levels has been reported to significantly decrease cell viability, growth, sphere formation, and tumorigenicity in multiple cancer types (Wuebben et al., 2016; Wuebben and Rizzino, 2017). Furthermore, overexpression of SOX2 correlates to gemcitabine resistance in pancreatic cancer cells (Jia et al., 2019), as well as higher malignancy in glioblastoma, esophageal, breast, and prostate cancers (Annovazzi et al., 2011; Jia et al., 2011; Wuebben and Rizzino, 2017).

Despite the importance of FGF/FGFR signaling in the maintenance of cancer cell stemness, the underlying mechanisms are not fully understood. We tested the hypothesis that FGF/FGFR signaling influences pancreatic cancer stemness by regulating SOX2. We describe a FGFR/AKT/SOX2 signaling axis in regulating pancreatic cancer stemness by modulating the protein level as well as cellular localization of SOX2. Inhibition of the FGF/FGFR may provide a new approach for the treatment of SOX2-positive pancreatic cancers.

## Materials and Methods

### Plasmids and Reagents

The plasmids for *SOX2* shRNA and mammalian and lentivirus-mediated protein overexpression were previously reported (Herreros-Villanueva et al., 2013). The plasmids for FGFR knockdown were constructed using *pLKO-1* lentiviral expression vector and the detailed gRNA sequences are listed in Table 1. HA-tagged wild type AKT (*HA-AKT*), its dominant negative mutant version (K179M, *AKT-KD*) and HA-ubiquitin (*HA-Ub*) expression plasmids were acquired from Addgene. All chemical reagents and antibodies used in this study are commercially available. FGFR and AKT inhibitors were purchased from MCE (MedChemExpress), LLL12 was purchased from KareBay Biochem. The following primary antibodies were purchased from Cell Signaling Technology: P-AKT (CST, 4060#), AKT (CST, 4685#), P-STAT3 (CST, 9145#) STAT3 (CST, 9139#), P-FGFR (CST, 3476#), FGFR1 (CST, 9740#), FGFR2 (CST, 23328#), SOX2 (CST, 3579#/4900#), CD44 (CST, 3570#), α-Tubulin (CST, 3873#), GAPDH (CST, 2118#), Histon-H3 (CST, 4499#), FLAG (CST, 14793#), HA (CST, 3724#). Antibody against CD24 was from Abcam (ab123946).

**TABLE 1:** Summary of shRNA guide sequences used in this study.

*Sh-FGFR1-1*	5′-AGTGGCTTATTAATTCCGATACTC-3′
*Sh-FGFR1-2*	5′-AGTGGCTTATTAATTCCGATACTC-3′
*Sh-FGFR2-1*	5′-CCAACCTCTCGAACAGTATTCTC-3′
*Sh-FGFR2-2*	5′-GCACACACTTACAGAGCACAACTC-3′
*Sh-FGFR3*	5′-GTACTGTGCCACTTCAGTGTGCTC-3′
*Sh-FGFR4*	5′-TCCATGATCGTCCTGCAGAATCTC-3′
*Sh-SOX2-1*	5′-GTACAGTATTTATCGAGATAACTC-3′
*Sh-SOX2-2*	5′-CAGCTCGCAGACCTACATGAACTC-3′

### Cell Culture and Transfection

HEK293, HEK293T and PDAC cell lines L3.6, BxPC3, Panc1, PaTu8988T, and HPNE (an HTERT-immortalized normal pancreatic epithelial cell line) were obtained from American Type Culture Collection (ATCC). They were maintained under recommended culture conditions. Cells were detached from the plates using 0.25% Trypsin/EDTA (Gibco) and transferred to a new 6 well dishes 24 h before transfection. Plasmid and Lipofectamine 2000 (Invitrogen) were mixed in Opti-MEM (Gibco) medium and incubated for 10 min before transfection. The medium was changed 4–6 h after transfection.

### Lentiviral Packaging, Transduction, and Selection of Stable Cells

For Lentiviral packaging, HEK293T cells were transfected with *VSVG*, *Gag/Pol* and *pLKO-shRNA* at a ratio of 0.25:0.75:1 and cultured for 48 h. During this time, the medium was harvested twice (at 24 and 48 h, respectively). The medium was filtered using a 0.45 μm filter (Millipore) and stored in an ultra-cold storage freezer. The particles were added into the cell medium together with 8 μg/ml polybrene to infect the host cells. After 48 h, infected cells were selected for another 72 h with 2 μM Puromycin Dihydrochloride (Invitrogen) for gene silencing or 5 μM Blasticidin (Invitrogen) for gene overexpression.

### RNA Isolation and Real-Time PCR

Total RNA was extracted from the pancreatic cancer cells using Trizol reagent according to the manufacturer’s instructions. cDNA was synthesized using Prime Script RT Reagent Kit (TaKaRa). Real-time PCR was carried out with CFX96 Real-Time System (Bio-Rad) and SYBR Premix Ex Taq (TaKaRa). All values were normalized to *GAPDH*. The gene-specific primers used in this research are listed in Table 2.

**TABLE 2:** Summary of qPCR primers used in this study.

*FGFR1*	F	5′-AACCTGCCTTATGTCCAGATC-3′
	R	5′-AGAGTCCGATAGAGTTACCCG-3′
*FGFR2*	F	5′-TCTGCATGGTTGACAGTTCTG-3′
	R	5′-TCTTCATTCGGCACAGGATG-3′
*FGFR3*	F	5′-GTCGTGGAGAACAAGTTTGG-3′
	R	5′-ACACCTTGCAGTGGAACTC-3′
*FGFR4*	F	5′-CTGGCTTAAGGATGGACAGG-3′
	R	5′-CCACAGCGTTCTCTACCAG-3′
*SOX2*	F	5′-CACACTGCCCCTCTCAC-3′
	R	5′-TCCATGCTGTTTCTTACTCTCC-3′
*CD24*	F	5′-GCCCCAAATCCAACTAATGC-3′
	R	5′-ACGTTTCTTGGCCTGAGTC-3′
*CD44*	F	5′-TCTTCAACCCAATCTCACACC-3′
	R	5′-TCCTGTCCAAATCTTCCACC-3′
*CD133*	F	5′-GTGGATGCAGAACTTGACAAC-3′
	R	5′-ACCCTTTTGATACCTGCTACG-3′

### Immunofluorescence Staining and Imaging

Glass Bottom Cell Culture Dishes (NEST, 801002) were used to grow cells for immunofluorescence. Approximately 5,000 cells were plated into dish for 24 h before treatment. Immunofluorescence staining was carried out with the primary antibodies against SOX2 and/or CD24 at 10 μl/ml. Donkey Anti-Rabbit Alexa Fluor^®^ 488 (Abcam, ab150073) and Donkey Anti-Rabbit Alexa Fluor^®^ 647 (Abcam, ab150075) were used as secondary antibodies. DAPI (D1306, Thermo Fisher Scientific) was used for counterstaining of the nuclei. Confocal images were collected with a LeicaSP8 confocal and Suite-Advanced Fluorescent software.

### Protein Extraction

Whole cell lysates were prepared in cell lysis buffer supplemented with a protease inhibitor cocktail (Roche, Basel, Switzerland) and quantified using the Bradford assay (LEAGENE, PT0010). An equivalent of 50 μg protein was separated by Sodium Dodecyl Sulfate Poly-Acrylamide and then transferred to a polyvinylidene fluoride membrane for Western blot analysis. The cytoplasmic and nuclear protein extraction was performed using a Cytoplasmic nuclear extract kit (Beyotime, P0027). Cells were dissociated and harvested from 10 cm dish for fractionation according to the manual instructions. Alpha-Tubulin was used as a loading control for cytoplasmic protein and Histone-H3 for nuclear protein.

### Immunoprecipitation and Immunoblotting

HEK293 cells were transfected with *Flag-SOX2* and *HA-Ub* expression vectors as indicated. Reagents were added into the medium 24 h after transfection and cultured for another 18 h. MG132 (20 μM, MCE) was added 4 h before harvesting. The cells were washed twice with pre-chilled PBS and whole cell lysates were prepared in RIPA buffer (50 mM Tris, pH 7.4, 2 mM EDTA, 1% NP-40, 0.25% sodium deoxycholate, 150 mM NaCl) supplemented with protease inhibitor cocktail (Roche, Basel, Switzerland). For ubiquitination assay, lysis buffer was also freshly supplemented with 1 mM iodoacetamide and 10 mM NEM as recently described (Guo et al., 2020). Protein concentrations were determined using the Bradford assay (LEAGENE, PT0010). Cellular extracts (500 μg) were incubated with the indicated antibody-conjugated beads overnight at 4°C. After washing the beads, the immunocomplexes were subjected to western blot. Immunoreactive bands were detected using ChemiDoc XRS+ System (Bio-Rad). To determine the protein half-life, cells were plated at a density of 500,000/well in a 6 cm dish before treatment. Inhibitors of interest and Cycloheximide (CHX, 50 μg/ml, MCE) were added to the cell medium at the indicated concentrations. Cells were scrapped and proteins were isolated after different periods of treatment. The harvested proteins were used to perform a western blot. All of the quantified western blots were performed three times independently.

### Sphere Formation Assay

Sphere formation assay was performed essentially as previously described (Herreros-Villanueva et al., 2013). Briefly, the cells were suspended in cancer stemness inducing (CSI) medium, DMEM-F12 (+HEPES & L-glutamine, Gibco) supplemented with epidermal growth factor (EGF, 20 ng/ml), FGF2 (10 ng/ml), N2 Supplement (Gibco), B27 (Gibco), 50 μg/ml insulin (Sigma) and 0.4% BSA (Sigma) Serum-Free Supplement and seeded in Ultra-Low attachment 6-well plates (Corning) at a density of 30,000 cells/well. After 10 days of culture, spheres were counted and photographed. To determine the effect of FGFR inhibitor on sphere forming efficiency, AZD4547, or Dovitinib, was added at 72 h after the initiation of suspension culture in CSI medium.

### Mouse Xenograft Model

Male athymic nude mice (BALB/c background) were purchased from Beijing Vital River Laboratory Animal Technology Company. The mice were housed and maintained in laminar flow cabinets under specific-pathogen-free conditions. The mice were used when they were 8 weeks old in accordance with Wenzhou Medical University Institutional Animal Care Guidelines. Approval Number: 2018-296. For *in vivo* injection, cells were harvested and suspended in phosphate buffer saline (PBS). The cell number was assessed using trypan blue exclusion, the cells were then diluted to 2 × 10^3^, 2 × 10^4^ and 2 × 10^5^/ml and mixed with Matrigel (Corning^®^ Matrigel^®^) at the volume ratio of 1:1 immediately before injection. Cells were injected subcutaneously at a volume of 0.1 ml for each site. Each experimental group contains six animals, cells were injected subcutaneously into the back of the mice. The number of tumors was calculated after 3 weeks.

### Statistical Analysis

All *in vivo* experiments were randomized and blinded. In all *in vitro* experiments including Real-time PCR (qPCR), Thiazolyl Blue Tetrazolium Bromide (MTT) assay, quantification of sphere formation and densitometric analysis for Western blots are presented as the mean ± SD. Statistical analysis and graphs were generated using GraphPad Prism 7.0 software (GraphPad, San Diego, CA, United States). Statistical differences were calculated by an unpaired two-tailed *t*-test, and those showing no differences were calculated by a one-tailed *t*-test. The values of *p* < 0.05 were considered statistically significant.

## Results

### Pharmacological FGFR Inhibition Impairs Pancreatic Cancer Stemness

**FIGURE 1:**
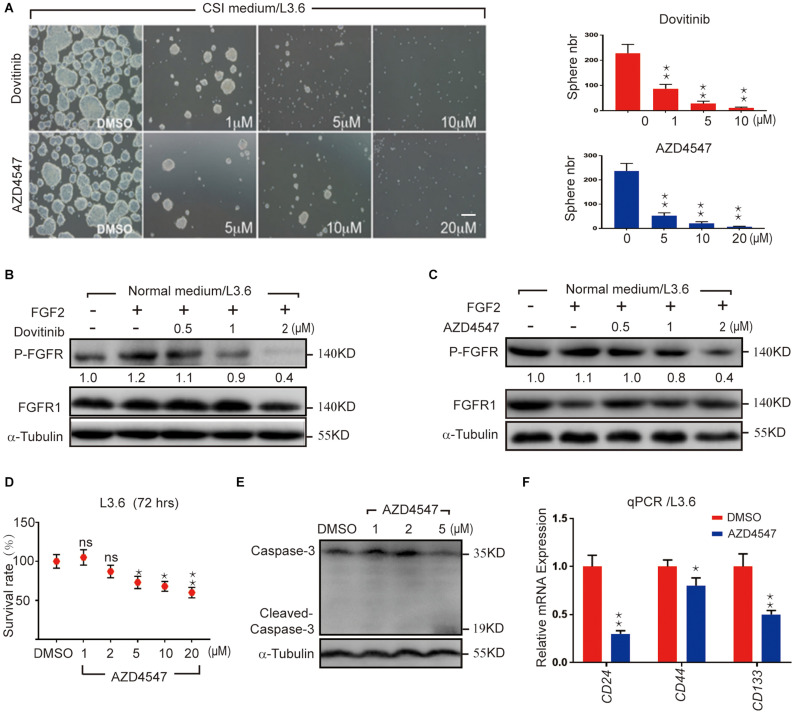
Pharmacological FGFR inhibition impairs pancreatic cancer stemness. **(A)** Sphere formation assay using L3.6 cells with different doses of FGFR inhibitors (AZD4547 and Dovitinib) and sphere number quantification from three independent experiments. scale bar: 200 μm. **(B,C)** Determination in L3.6 cells of FGF2-induced FGFR phosphorylation by western blot in the presence of AZD4547 or Dovitinib. FGFR1 and alpha-Tubulin are used as loading controls. Numbers below the blots are quantifications for three independent experiments. **(D)** Cell survival rate after 72 h of treatment with different doses of AZD4547 or DMSO control. **(E)** Determination by western blot of L3.6 cells of cleaved Caspase 3 in the presence of AZD4547. **(F)** Expression of stemness markers *CD24*, *CD44*, and *CD133* by qPCR in PDAC with and without AZD4547 treatment. *p ≤ 0.05, **p ≤ 0.01.

FGF/FGFR signaling is crucial for organogenesis and tissue regeneration, FGF is also a constituent of the CSI medium for spheroid formation, a surrogate assay for stemness in cancer cells. To test the hypothesis that FGF/FGFR pathway regulates PDAC cancer stemness, L3.6 cells were treated with Dovitinib, a pan-RTK inhibitor and AZD4547, a reversible FGFR specific inhibitor, respectively. Spheroid-forming assay was performed to determine stemness potential. Here, Dovitinib was applied as a positive control. A reproducible number of spheres was induced and quantified from 30,000 L3.6 cells/well (plated in 6 wells dish). In our experimental conditions, around 230 spheroids were systematically observed in the control medium (228 ± 35, *n* = 3). Both of these inhibitors significantly inhibited the ability of L3.6 cells to form spheres. Dovitinib significantly inhibited sphere formation at 1 μM (87 ± 9 vs. 228 ± 35, *p* < 0.01, *n* = 3, for treated vs. control, respectively). A similar effect was observed with AZD4547 but at a higher dose (5 μM) (52 ± 6 vs. 237 ± 31, *p* < 0.01, *n* = 3) (Figure 1A and corresponding quantification). Both Dovitinib and AZD4547 (at 1–2 μM range) dramatically inhibited FGFR phosphorylation (Figures 1B,C). Overall, our results indicate that inhibition of the FGFR signaling effectively decreases the sphere-forming ability of PDAC cells. To clarify whether this decrease in sphere-forming efficiency is stemness-related or simply due to an increase in cell death, the MTT assay and western blot analysis were performed. Our results indicated that the survival of L3.6 cells decreased in a dose dependent manner. However, this decrease was not significantly different between control and 2 μM of AZD4547 (100.0% ± 0.1 vs. 88.0% ± 0.1, *p* > 0.05, *n* = 6), the dose that was used in the follow up experiments (Figures 1D,E). These results indicate that the decrease in sphere number was the result of a loss in stemness rather than an increase in cell death. We further compared the mRNA level of cancer stemness markers in AZD4547-treated (2 μM) vs. non-treated control L3.6 cells and found that *CD24*, *CD44*, and *CD133*, were dramatically down-regulated upon AZD4547 treatment (Figure 1F). Together, these results indicate that FGFR inhibition attenuates cancer stemness.

### Suppression of FGFR Expression Reduced Stemness in *vitro* and Tumor Formation in *vivo*

**FIGURE 2:**
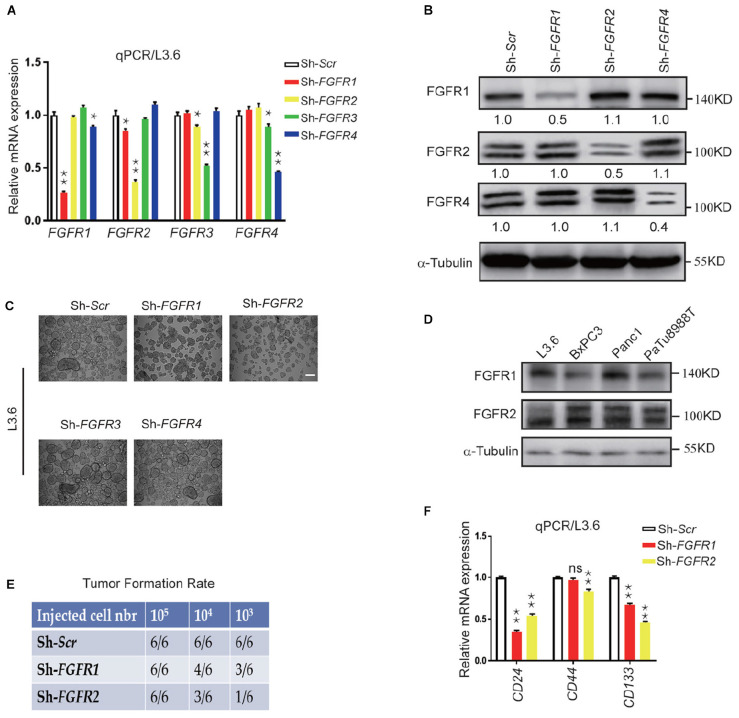
Genetic silencing of FGFR expression leads to reduced stemness *in vitro* and tumor formation *in vivo*. **(A,B)** Expression of *FGFRs* by qPCR and western blot in L3.6 cells upon silencing specific FGFRs. Numbers below the blots are quantifications for three independent experiments. **(C)** Sphere formation assay in L3.6 cells following specific FGFR knockdown and quantification of sphere numbers for three independent experiments. Scale bar: 200 μmm. **(D)** FGFR1 and FGFR2 protein expression by western blot in several pancreatic cancer cell lines. **(E)** Tumor formation rate 3 weeks following the subcutaneous inoculation of different numbers of L3.6 cells to nude mice. **(F)** Expression of stemness markers *CD24*, *CD44*, and *CD133* by qPCR in L3.6 cells upon silencing of *FGFR1* or *FGFR2*. *p ≤ 0.05, **p ≤ 0.01.

FGF ligands signal via four specific receptors in humans (FGFR1 to FGFR4). In order to check the role of each FGFR in governing the cancer cell stemness, lentiviral shRNA expression vectors targeting each FGFR was used to knockdown endogenous FGFRs. The specific effect of each lentiviral construct was validated by qPCR and western blot on L3.6 cells. Although FGFR3 was not detected by western blot in L3.6, but it can be detected at mRNA level, we presume there might be a very low level of FGFR3 expression in L3.6 cells (Figures 2A,B). Sphere-forming assays were then performed to detect the effect of each FGFR silencing on stemness in L3.6 cells. We found that suppression of either FGFR1 or FGFR2 significantly decreased the number of spheres compared to the scramble control (78 ± 13 vs. 253 ± 24, *p* < 0.01, *n* = 3 and 53 ± 11 vs. 253 ± 24, *p* < 0.01, *n* = 3, for *FGFR1* and *FGFR2* silencing, respectively). Interestingly, interfering with FGFR3 or FGFR4 did not affect sphere formation (239 ± 33 vs. 253 ± 24, *p* > 0.05, *n* = 3 and 226 ± 26 vs. 253 ± 24, *p* > 0.05, *n* = 3, for *FGFR3* and *FGFR4* silencing, respectively) (Figure 2C and quantification). The sizes of the spheres were also measured (Supplementary Figure S1A), and this showed no significant differences among different groups. These results suggest that FGFR1 and FGFR2, play important roles in PDAC cell stemness. Of note, according to the cancer genome atlas database, FGFR3 is barely expressed in the pancreas or in pancreatic cancer (Mohammadi et al., 2005) which has also been proven by qPCR.

We then examined the expression of FGFR1 and FGFR2 by western blot and showed that FGFR1 and FGFR2 are widely expressed throughout these pancreatic cancer cell lines (Figure 2D). We also carried out a xenograft efficiency assay using L3.6*-ShScr*, L3.6*-ShFGFR1*, and L3.6*-ShFGFR2* cell lines (Figure 2E). A significant reduction in tumor formation rates at the two lower numbers of cancer cells injected was observed in the knockdown groups. Furthermore, the growth of the knocking down cells was slower than that of control ones according to the kinetic curves of tumor growth (Supplementary Figure S1B). Upon examining the expression of stemness markers in *Sh-Scr, Sh-FGFR1*, and *Sh-FGFR2* cell lines, we found that compared to *Sh-Scr* cell line, the *Sh-FGFR1* and *Sh-FGFR2* cell lines displayed much lower expression of *CD24* (0.35 ± 0.02 vs. 1 ± 0.03, *p* < 0.01, *n* = 3 and 0.54 ± 0.02 vs. 1.00 ± 0.02, *p* < 0.01, *n* = 3, for *FGFR1* and *FGFR2* silencing, respectively) and *CD133* (0.67 ± 0.02 vs. 1.00 ± 0.03, *p* < 0.01, *n* = 3 and 0.46 ± 0.02 vs. 1.00 ± 0.02, *p* < 0.01, *n* = 3 for *FGFR1* and *FGFR2* silencing, respectively) (Figure 2E). Our results suggest that FGFR1 and FGFR2, but not FGFR3 or FGFR4, are involved in the stemness regulation of PDAC.

### SOX2 Expression Correlates With Stemness and Its Silencing Leads to Decreased Spheroid Formation

**FIGURE 3:**
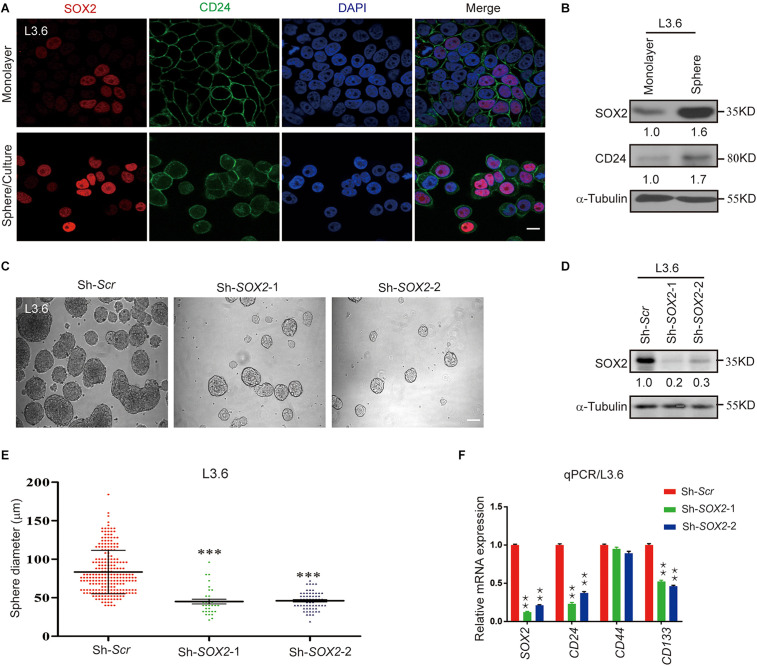
SOX2 expression in L3.6 cells correlates with stemness and silencing of *SOX2* expression leads to decreased spheroid formation. **(A)** Monolayer and spheroid L3.6 cells were stained with DAPI, SOX2 and CD24. Scale bar: 20 μm. **(B)** Expression of SOX2 by western blot in monolayer and spheroid L3.6 cells. Numbers below the blots are quantifications for three independent experiments. **(C)** Sphere formation assay using SOX2 knockdown in L3.6 cell lines compared with scramble control. Scale bar: 200 μm. **(D)** Western blot was performed to detect the expression of SOX2 in *SOX2* knockdown L3.6 cells compared to scramble control. Numbers below the blots are quantifications for three independent experiments. **(E)** Quantification of sphere diameter in scramble control vs. *SOX2* knockdown (each spot represents one sphere). **(F)** Expression of stemness markers *CD24*, *CD44* and *CD133* by qPCR in L3.6 cells upon silencing of *SOX2.* **p ≤ 0.01, ***p ≤ 0.001.

As previously mentioned, SOX2 is a key transcription factor that induces stemness in cancer (Wuebben and Rizzino, 2017). Immunofluorescence was performed to detect the expression of SOX2 in L3.6 cells grown as a monolayer or as spheres. In monolayer cells, the expression of the cancer stemness marker CD24 was relatively concentrated on the membrane in spheroid cells and SOX2 expression was mostly confined in the nucleus (Figure 3A and Supplementary Figure S2A). Western blot of SOX2 and CD24 showed that both proteins were upregulated in spheroid cells (Figure 3B). To identify the relevance of SOX2 to cancer stemness, we engineered two stable cell lines designated as *Sh-SOX2*-1 and *Sh-SOX2*-2, which display a knockdown of *SOX2* and Sh-scramble control (Figure 3C). We tested these two cell lines and the corresponding control in the sphere-forming assay. Our results indicate that *SOX2* knockdown cell lines exhibits a significant decrease in sphere-forming capacity when compared to scramble control cells (42 ± 6 vs. 237 ± 31, *p* < 0.01, *n* = 3 and 78 ± 12 vs. 237 ± 31, *p* < 0.01, *n* = 3, for *Sh-SOX2*-1 and *Sh-SOX2*-2 lines, respectively). Additionally, the average diameter of the spheroids also decreased (46 μm ± 6 vs. 82 μm ± 24, *p* < 0.01, *n* = 3 and 50 μm ± 3 vs. 82 μm ± 26, *p* < 0.01, *n* = 3, for *Sh-SOX2*-1 and *Sh-SOX2*-2 lines, respectively) (Figures 3D,E). Finally, the expression of *CD24* in these two cell lines was greatly reduced compared to control cells (0.23 ± 0.01 vs. 1.00 ± 0.01, *p* < 0.01, *n* = 3, 0.21 ± 0.01 vs. 1.00 ± 0.01, *p* < 0.01, *n* = 3, for *Sh-SOX2*-1 and *Sh-SOX2*-2 lines, respectively) (Figure 3F). A similar result was observed for *CD133* (0.52 ± 0.01 vs. 1.00 ± 0.01, *p* < 0.01, *n* = 3, 0.46 ± 0.01 vs. 1.00 ± 0.01, *p* < 0.01, *n* = 3, for *Sh-SOX2*-1 and *Sh-SOX2*-2 lines, respectively). The protein levels of CD24 and CD133 were further detected using immunoblot. The result again proves that SOX2 knockdown leads to the down regulation of both stemness markers in pancreatic cancer (Supplementary Figure S2B).

### *SOX2* Overexpression Leads to Increase Spheroid Formation, a Process Inhibited by AZD4547

**FIGURE 4:**
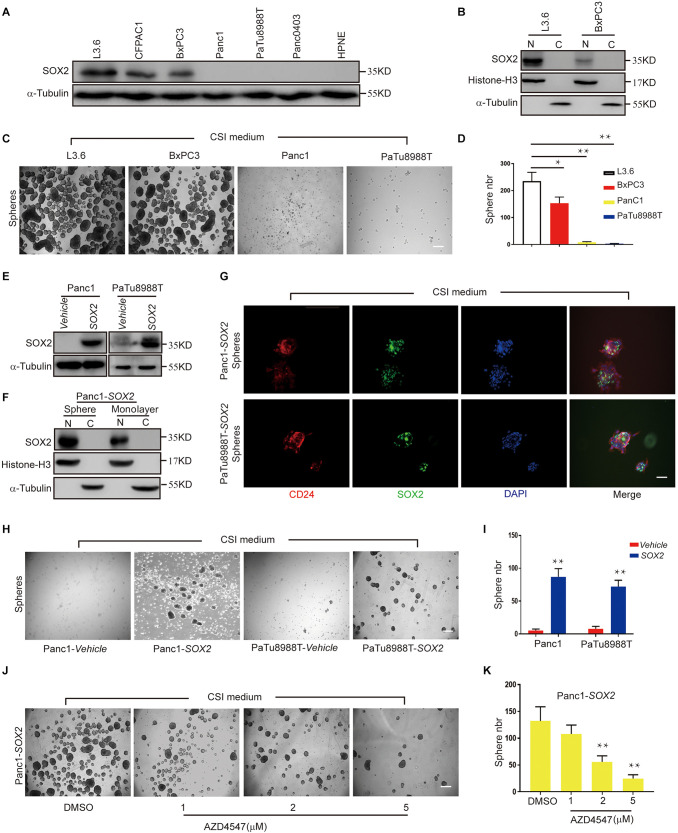
*SOX2* overexpression in SOX2 negative PDAC lines leads to increased spheroid formation. **(A)** Western blot for SOX2 showing SOX2-positive and SOX2-negative pancreatic cell lines. Alpha-Tubulin is used as a loading control. **(B)** SOX2 detection in cytoplasm and nucleus protein fractions. Histone-H3 and alpha-Tubulin are used as compartment specific loading controls. **(C)** Sphere formation assay using SOX2-positive and SOX2-negative PDAC lines. **(D)** Corresponding sphere number quantification for three independent experiments, *p ≤ 0.05, **p ≤ 0.01. **(E)** Validation by western blot of that SOX2 overexpression has been achieved in the two pancreatic cancer cell lines with low/negative endogenous SOX2 expression. **(F)** Expression of SOX2 in monolayer and spheroid Panc1-*SOX2* cells, separated into the nuclear and cytoplasmic fractions. **(G)** Validation of SOX2 overexpression and localization in the two PDAC lines by immunofluorescence. Spheroids were stained with SOX2 and CD24 specific antibodies, respectively, and counterstained with DAPI. Scale bar: 100 μm. **(H)** Sphere formation of previous SOX2-negative cells (Panc1 and PaTu8988T) transfected with either vehicle plasmid (control) or SOX2-plasmid (experimental). Note that SOX2 overexpression is sufficient to increase sphere formation. **(I)** Corresponding sphere number quantification for three independent experiments. **(J)** Impact of AZD4547 treatment on SOX2-overexpressing Panc1 cell ability to form spheres. **(K)** Corresponding sphere number quantification for three independent experiments. Scale bar for **(C,H,J)**: 200 μm, scale bar for **(H)**: 100 μm.

We previously reported that SOX2 is expressed in less than a quarter of primary PDAC tissues and only certain pancreatic cancer cell, and its expression positively correlates with sphere-forming potential (Herreros-Villanueva et al., 2013). When we further examined the expression of SOX2 in a panel of available PDAC cell lines, consistent with our previous observations, we found SOX2 is abundantly expressed in L3.6 cells and also readily detected in CFPAC1 and BxPC3 cells. However, in Panc1, PaTu888T, Panc0403, and HPNE (normal pancreatic tissue cells), the expression of SOX2 was minimal to undetectable (Figure 4A). When cytoplasmic and nuclear protein fractions were isolated and tested for SOX2 localization, we found that SOX2 was almost exclusively present in the nucleus and that the concentration of SOX2 in L3.6 cells was higher than that in BxPC3 cells (Figure 4B). We then investigated the capacities of the two cell lines (L3.6 and BxPC3) to express endogenous SOX2. We also looked at the spheroid-forming capacity of two other cell lines with low/negative endogenous SOX2 (Panc1 and PaTu8988T). As shown in Figures 4C,D, the number of spheres with L3.6 cells was higher than the one obtained with BxPC3 cells. On the other hand, spheres were barely detectable in Panc1 and PaTu8988T cell lines. These results demonstrate a positive correlation between endogenous SOX2 expression and sphere formation potential in PDAC cells.

To further determine the role of SOX2 in PDAC stemness, we overexpressed *SOX2* in Panc1 and PaTu8988T using engineered lentiviruses (Vehicle control and SOX2 overexpression) and obtained stable SOX2 expression with their corresponding control lines (Figure 4E). The expression and localization of SOX2 in monolayer and spheroid made with Panc1*-SOX2* and PaTu8988T*-SOX2* was confirmed by immunoblot analysis (Figure 4F). Immunofluorescence was also applied to check the expression of SOX2 and stemness marker CD24 (Figure 4G). Our results show that SOX2 expression is up-regulated in spheres and is mainly present in the nuclei. Significantly, we further observed that SOX2 overexpression alone is sufficient for Panc1 and PaTu8988T cells to gain sphere-forming capabilities compared to control lines (Figures 4H,I), which is consistent with Villanueva’s earlier work (Herreros-Villanueva et al., 2013). This again re-enforces our conclusion that SOX2 is a key regulator of cancer stemness in PDAC. Spheroid assays were further performed in Panc1*-SOX2* cells after treatment with AZD4547. We found that Panc1*-SOX2*, like L3.6 cells treated under the same conditions, displayed a decrease in sphere number of around 60% upon AZD4547-treatment (2μM, 56 ± 12 vs. 132 ± 36, *p* < 0.01, *n* = 3) (Figures 4J,K). MTT assay was carried out to test whether AZD4547 inhibits the monolayer growth of the parental Panc1 and PaTu8988T cells. It turns out that AZD4547 does not significantly inhibit the cell growth in these two cell lines within 5 μM (Supplementary Figures S2C,D). Considering exogenous and endogenous SOX2 can both be regulated, we hypothesize that SOX2 might be regulated in the protein level upon FGFR inhibition.

### FGFR Inhibition Leads to SOX2 Degradation

**FIGURE 5:**
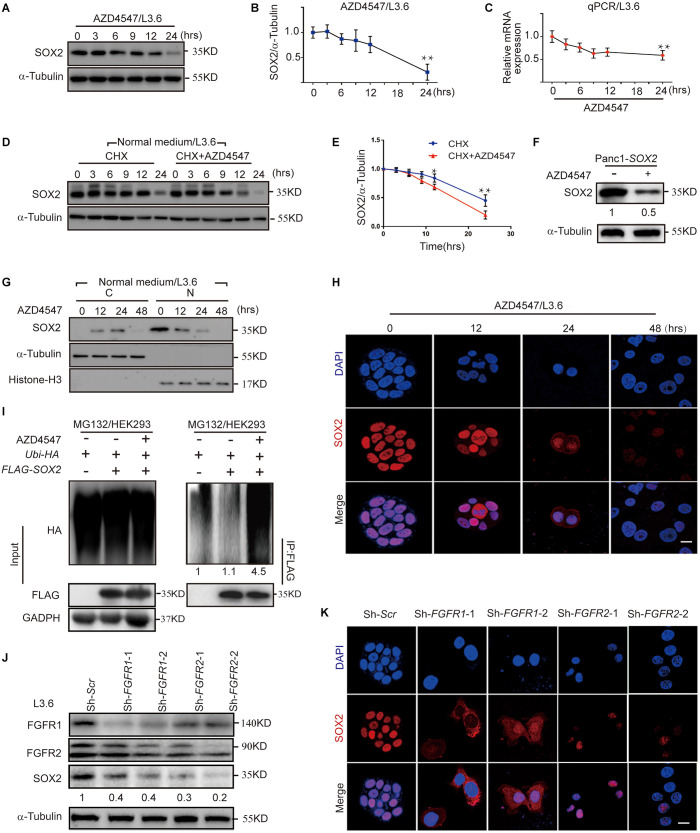
FGFR inhibition leads to SOX2 degradation. **(A)** Western blot for SOX2 upon AZD4547 (2 μM) treatment at different time points. Alpha-Tubulin was used as a loading control. **(B)** Corresponding SOX2 quantification for three independent experiments. **(C)** Quantification of *SOX2* mRNA levels at different time points following AZD4547 (2 μM) treatment. **(D)** Western blot was used to detect SOX2 expression upon treating with CHX (50 μg/ml) alone or in combination with AZD4547 (2 μM) at different time points. **(E)** Corresponding quantification of SOX2 for three independent experiments. **(F)** Western blot was used to detect SOX2 upon AZD4547 (2 μM) treatment after 24 h in *SOX2*-overexpressing cell line. **(G)** SOX2 expression in the nuclear and cytoplasmic fractions at different time points upon AZD4547 (2 μM) treatment. **(H)** SOX2 expression by immunofluorescence in L3.6 cells upon AZD4547 (2 μM) treatment at different time points. **(I)** Ubiquitination analysis of SOX2 in HEK293 cells with or without AZD4547 (2 μM) in presence of MG132 (20 μM) treatment to block degradation. Numbers below the blots are quantifications for the blots. **(J)** Western blot was used to detect FGFR1, FGFR2, and SOX2 expression upon *FGFR1* or *FGFR2* knockdown in L3.6 cells. Numbers below the blots are quantifications for three independent experiments. **(K)** SOX2 expression by immunofluorescence upon *FGFR1* or *FGFR2* knockdown in L3.6 cells. *p ≤ 0.05, **p ≤ 0.01, scale bar: 20 μm.

Inactivation of FGFR signaling or knockdown of SOX2 significantly inhibited PDAC stemness, suggesting a functional connection between FGFR signaling and SOX2. In L3.6 cells treated with FGFR inhibitor AZD4547, SOX2 protein levels began to diminish at around 6 h and decreased significantly at 24 h (Figures 5A,B). However, only a modest decrease in *SOX2* mRNA level was observed in AZD4547-treated cells when compared to the control at 24 h (Figure 5C). These results demonstrate that inhibition of FGFR signaling impacts SOX2 expression not only at the transcriptional level, but more significantly, at the post-transcriptional level. To test if SOX2 can be regulated at the protein level, we determined the half-life of protein of interests with cycloheximide (CHX) treatment (Schneider-Poetsch et al., 2010). We found that, upon CHX-mediated protein synthesis inhibition, AZD4547 treatment could accelerate the degradation of SOX2 in L3.6 cells (0.62 vs. 0.78, *p* < 0.05, *n* = 3) (Figures 5D,E). Further examination on Panc1*-SOX2* cells also indicates a decrease of SOX2 protein level upon AZD4547 treatment (Figure 5F). Considering that the transcription of SOX2 is stable in Panc1*-SOX2* cells, the most logical possibility is that AZD4547 impacts SOX2 levels through post-translational modifications. We therefore further determined the presence of SOX2 in the cytoplasm and nucleus after AZD4547 treatment by using western blot and immunofluorescence. Although SOX2 is exclusively detected in the nucleus of L3.6 cells under normal culture conditions, treatment with AZD4547 elicited SOX2 cytoplasmic translocation beginning at 12 h. Remarkably, both nuclear and cytoplasmic SOX2 were decreased to barely detectable levels after 48 h (Figures 5G,H). We propose that, in L3.6 cells, AZD4547 promotes SOX2 nuclear export to the cytoplasm and leads to its further degradation.

To further examine the effects of FGFR inhibition on SOX2 degregation, we then performed ubiquitination assays. Our resuts indicated that SOX2 ubiquitination was significantly increased after inhibiting FGFR signaling (Figure 5I). The same experiment was carried out in L3.6 cells and showed similar results (Supplementary Figure S3A). Immunofluorescence and immunblot were performed to confirm the specificity of this regulation. We observed a significant change both at the protein level and in the intracellular localization of SOX2 in *FGFR1* and *FGFR2* knockdown cell lines (*Sh-FGFR1*-1, *Sh-FGFR1*-2, *Sh-FGFR2*-1, and *Sh-FGFR2*-2). This demonstrates that FGFR1 and FGFR2 inhibition is causative for SOX2 down-regulation (Figures 5J,K). Ubiquitination analysis was also performed in the knockdown cell lines, which showed that SOX2 ubiqitination is increased in FGFR1 and FGFR2 knockdown cell lines (Supplementary Figure S3B). Next, we applied western blot to detect the effect of FGFR1 and FGFR2 double knockdown on SOX2 regulation, which revealed a stronger down-regulation of SOX2 in double knockdown cells (Supplementary Figure S3C).

### FGFR Regulates SOX2 Mainly Through AKT Pathway

**FIGURE 6:**
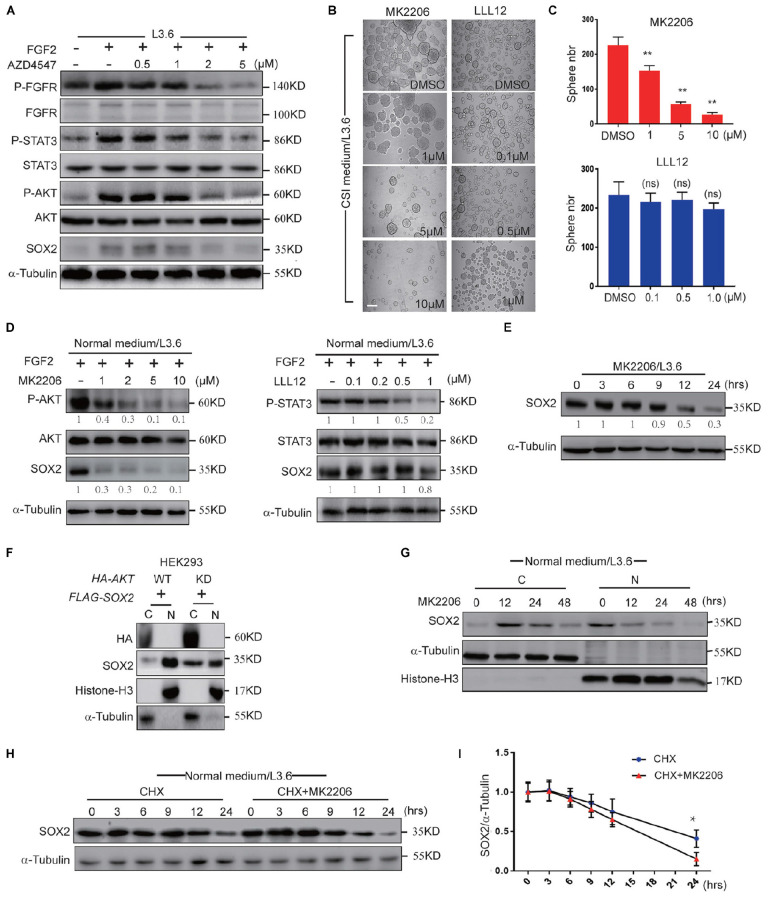
FGFR regulates SOX2 mainly through AKT. **(A)** Western blot analysis of key FGFR downstream pathways in L3.6 cells treated with different doses of AZD4547 together with FGF2 (10 ng/ml) for 12 h. **(B)** Sphere formation assay using L3.6 cells treated with different doses of MK2206 (AKT inhibitor) and LLL12 (STA3 inhibitor). Scale bar: 200 μm. **(C)** Corresponding sphere number quantification for three independent experiments. **(D)** Western blot analysis of pathway inhibition efficiency of MK2206 and LLL12 for 24 h. Numbers below the blots are quantifications for three independent experiments. **(E)** Western blot was performed to quantify SOX2 expression levels upon MK2206 (2 μM) treatment in L3.6 cells at indicated time points. Numbers below the blots are quantifications for three independent experiments. **(F)** SOX2 detection in cytoplasmic and nuclear fractions upon transfection with *AKT*-WT or *AKT*-KD in HEK293. **(G)** SOX2 detection in cytoplasmic and nuclear fractions upon MK2206 (2 μM) treatment at different time points. **(H)** Western blot was carried out to quantify SOX2 expression level upon treating with CHX (50 μg/ml) with and without MK2206 (2 μM) in L3.6 cells at different time points. **(I)** Corresponding quantification for three independent experiments. *p ≤ 0.05, **p ≤ 0.01.

FGFR can activate multiple downstream pathways, among which the STAT3 pathway and AKT pathway have been reported to be closely related to cancer stemness (Dong et al., 2014; Zhang et al., 2016). FGF2 is a member of the FGF family that is highly expressed in the adult pancreas (Kornmann et al., 1998) and can bind and activate all four FGF receptors (Ornitz and Itoh, 2015). We used western blot to determine the activation of the STAT3 and AKT pathways following treatment by FGF2 and AZD4547 in L3.6 cells. Our results showed that the levels of phosphorylated STAT3 and AKT were significantly increased after FGF2 stimulation and down-regulated following AZD4547 treatment (Figure 6A), suggesting that FGF/FGFR signaling can activate both the AKT and STAT3 pathway in PDAC. Spheroid assays were then carried out to determine the effect of these two pathways on PDAC stemness. MK2206 (Zhou et al., 2015) and LLL12 (Nie et al., 2018) are AKT and STAT3 inhibitors, respectively. Our results showed that sphere formation was dramatically decreased (about 75%) upon AKT inhibitor MK2206 treatment (2μM) (57 ± 7 vs. 226 ± 24, *p* < 0.01, *n* = 3), but did not change significantly with the STAT3 inhibitor LLL12 (1 μM) (198 ± 15 vs. 234 ± 33, *p* > 0.05, *n* = 3) (Figures 6B,C). Further supporting our functional relationship with SOX2, our western blot and qPCR results indicated that both inhibitors were capable of blocking the phosphorylation of their respective targets. Interestingly, AKT inhibition down-regulated SOX2 while STAT3 inhibition did not (Figure 6D and Supplementary Figure S3D). We therefore propose that, downstream of FGFR signaling, AKT regulates cancer stemness via impacting SOX2 ubiquitination and subcellular localization.

Next, we tested the function of AKT inhibition under multiple conditions. First, the expression of SOX2 in L3.6 cells was monitored by western blot following MK2206 treatment. The protein level of SOX2 was dramatically reduced upon AKT inhibition (Figure 6E). We then tested the function of AKT on overexpressed SOX2. *FLAG-SOX2* was co-transfected with HA-tagged wild type AKT (*AKT-HA*) or kinase-dead AKT (*AKT-KD*) and the expression of SOX2 in the cytoplasmic and nuclear fractions was examined by immunoblot. Nuclear SOX2 was decreased when HEK293 cells were transfected with *AKT-KD* compared to *AKT-HA* (Figure 6F). The analysis of the cytoplasmic and nuclear fractions indicated that SOX2 expression was increased in the cytoplasm at 12 h and markedly decreased at 48 h following MK2206 treatment (Figure 6G). CHX assay was used to determine the effect of MK2206 on the half-life of SOX2. The results indicated that MK2206 treatment accelerated the degradation of SOX2 after protein synthesis inhibition (0.64 ± 0.03 vs. 0.75 ± 0.05, *p* < 0.05, *n* = 3) (Figures 6H,I). Therefore, AKT inhibition largely recapitulated the effect of FGFR inhibition in promoting SOX2 cytoplasmic translocation and degradation, suggesting that AKT participates in mediating the effect of FGFR on SOX2 regulation.

## Discussion

The association of FGFs with cancer stemness has been widely accepted but, to our knowledge, there has not been detailed analysis of its mechanism. It particularly remains unclear as to what are the nuclear mediators of FGF that promote cancer stemness. In this study, we combined the use of pharmacological inhibitors and genetic manipulations to modulate the activity and expression of different FGFRs as well as the downstream mediators such as AKT and SOX2. We employed the sphere-formation assay as a surrogate approach to evaluate the impact of such manipulations on self-renewal capabilities *in vitro* (Lee et al., 2017) and further used the xenograft model to evaluate their tumorigenic properties *in vivo* (Zhao et al., 2019). Though the regulation of FGF2 and FGFR2 on SOX2 has been already reported in the development phase (Mansukhani et al., 2005), we were the first to demonstrate that FGFR signaling regulates the protein stability of SOX2 through AKT and promotes its nuclear localization, thus enhancing stemness in pancreatic cancer. In addition, we also showed that FGFR1 and FGFR2 are key receptors in regulating pancreatic cancer stemness.

CSCs play critical roles in resistance to anti-cancer treatment and are responsible for metastasis in several human malignancies, including PDAC (Valle et al., 2018). CSCs exhibit several characteristics such as enhanced invasive properties and metastasis, drug resistance and immune tolerance that make them difficult to eradicate with conventional therapy. In pancreatic cancer, one of the most common drugs used is gemcitabine, a DNA-damaging compound. However, the therapeutic effect of gemcitabine or its combination with nab-paclitaxel is still far from satisfactory (Shi et al., 2012; Von Hoff et al., 2013). An important reason for the failure of gemcitabine treatment is the acquired resistance of PDAC cells following the treatment. Indeed, it was reported that cancer stemness was increased when patients were treated with gemcitabine (Zhang et al., 2016). Additionally, even in *KRAS* mutated pancreatic cancer cells, such as L3.6 cells, the inhibition of FGFR activity is still capable of reducing stemness. We therefore propose that FGFR inhibitors could potentially be ancillary drugs that inhibit cancer stemness, thereby enhancing the therapeutic effect of other anti-cancer drugs.

The regulation of cancer stemness is a complicated process. SOX2 is considered to be a key factor in the regulation of this process. It has been demonstrated in numerous cancers that there is cell-to-cell variation in the expression of SOX2, even in the same tumor (Wuebben and Rizzino, 2017). In our experiments, SOX2-deficient pancreatic cancer cells failed to form spheres. Moreover, SOX2 overexpression in SOX2-low/negative cells was sufficient induce sphere formation. As sphere-forming ability is related to self-renewal capacity, cells that present higher sphere-forming capacities normally display a higher degree of tumor formation and metastasis, both quantitatively and qualitatively in xenograft experiments (O’Brien et al., 2010). Our results showed that interfering with *SOX2* expression in SOX2-expressing cells significantly reduces their cancer stemness. SOX2 expression in these cells is therefore necessary to maintain stemness and can be a promising target for the treatment of SOX2-positive cancers. However, as SOX2 is normally located in the nucleus where it acts as a transcription factor, SOX2 inhibition is difficult to achieve through the use of small molecular compounds.

Though earlier work has already shown that FGF2 and FGFR1 nuclear translocation in pancreatic leads to cell invasion (Coleman et al., 2014). Our work is focused on the stemness regulation of FGF/FGFR signaling in pancreatic cancer. Our study shows that FGFRs play important roles in SOX2 protein stabilization and nuclear localization in cancer stemness regulation. Interestingly, the inhibition of FGFRs not only inhibits the function of endogenous SOX2, but also strongly inhibits the function of exogenously overexpressed SOX2. Though studies have already shown that SOX2 induction by FGF and FGFR2 activation inhibits osteoblast differentiation (Mansukhani et al., 2005), we propose that FGFR inhibition can also be an effective scheme for indirect inhibition of SOX2 in pancreatic cancer.

As one of the main pathways downstream of FGFR, AKT has already been reported to associate with SOX2. Overexpression of SOX2 protected PDAC cells from the growth inhibitory effects of AKT inhibitors; whereas, knocking down SOX2 enhanced the inhibition in the presence of the inhibitors (Wuebben and Rizzino, 2017). AKT also promote its nuclear localization in breast cancer (Schaefer et al., 2015; Wang et al., 2019). Our study also indicates that the FGFR/AKT axis is not only capable of maintaining nuclear localization of SOX2, but also stabilizes the SOX2 protein through the ubiquitination modification, thus enhancing SOX2 function in pancreatic cancer cells. Based on the sequence analysis, the human SOX2 protein sequence contains an AKT recognition motif (RPRRX-S/T) and a predicted phosphorylation site (Thr116 in human, Thr118 in mouse). Interaction of AKT with SOX2 has been reported to promote its stabilization through phosphorylation at Thr118, which enhances the transcriptional activity of SOX2 in pluripotent stem cells (Jeong et al., 2010). A more recent study on esophageal cancer also showed that AKT-phosphorylation promotes SOX2 stabilization by preventing its ubiquitination and degradation by UBR5. AKT inhibitor can effectively downregulate SOX2 and suppress cancer stemness (Wang et al., 2019). In our study, we discovered that the ubiquitination and subcellular localization of SOX2 are both impacted by AKT activity down-stream of FGFR signaling. Though not as significant as the protein level, FGF/FGFR inhibition also reduced the mRNA level of SOX2, indicating that there might be an additional regulatory mechanism at the transcriptional level. Along this line, Rizzino’s group has previously reported a AKT-mediated negative feedback transcriptional control loop between SOX2 and FOXO1 that contributes to a tight regulation of self-renewal in pluripotent stem cells (Ormsbee Golden et al., 2013).

Besides SOX2, there are also other factors mediating cancer stemness in pancreatic cancer (Herreros-Villanueva et al., 2014; Ercan et al., 2017). For example, it has been shown that cells displaying low SOX2 expression, such as Panc1 and Patu8988T, also presented stem-like cell properties and were still capable of tumor initiation and metastasis in xenograft even though they presented lower self-renewal capability in sphere-forming assay (Zhao et al., 2019). Therefore, the expression of SOX2 in tumors should be confirmed before starting combined therapy with FGFR or AKT inhibitors. In conclusion, we report that an FGFR/AKT/SOX2 signaling axis controls cancer stemness in PDAC and may therefore represent a potential therapeutic target in the fight against this very aggressive form of cancer.

## Data Availability Statement

The datasets generated for this study are available on request to the corresponding author.

## Ethics Statement

The animal study was reviewed and approved by Wenzhou Medical University Institutional Animal Care Guidelines. Approval Number: 2018-296.

## Author Contributions

M-YQ and J-SZ: conceptualization. M-YQ, QG, RY, JB, and LD: methodology. M-YQ, QG, JL, RY, JB, WW, YC, RH, Y-QL, and LD: data acquisition and analysis. DB, ZL, and SB: resources. M-YQ, DB, ZL, SB, and J-SZ: manuscript writing and revision. XL and J-SZ: study supervision and funding of the project.

## Conflict of Interest

The authors declare that the research was conducted in the absence of any commercial or financial relationships that could be construed as a potential conflict of interest.
