# Corrigendum: An FGFR/AKT/SOX2 Signaling Axis Controls Pancreatic Cancer Stemness

**DOI:** 10.3389/fcell.2020.594589

**Published:** 2020-10-02

**Authors:** Mei-Yu Quan, Qiang Guo, Jiayu Liu, Ruo Yang, Jing Bai, Wei Wang, Yaxin Cai, Rui Han, Yu-Qing Lv, Li Ding, Daniel D. Billadeau, Zhenkun Lou, Saverio Bellusci, Xiaokun Li, Jin-San Zhang

**Affiliations:** ^1^School of Pharmaceutical Sciences and International Collaborative Center on Growth Factor Research, Wenzhou Medical University, Wenzhou, China; ^2^Institute of Life Sciences, Wenzhou University, Wenzhou, China; ^3^Center for Precision Medicine, The First Affiliated Hospital of Wenzhou Medical University, Wenzhou, China; ^4^Division of Oncology Research and Schulze Center for Novel Therapeutics, Mayo Clinic, Rochester, MN, United States; ^5^Cardio-Pulmonary Institute, Member of the German Lung Center, Justus Liebig University Giessen, Giessen, Germany

**Keywords:** FGFR, SOX2, pancreatic cancer, stemness, sphere-formation assay

In the original article, there was a mistake in Figures 2C, 6B, and S3C as published. The control tubulin immunoblot shown in Figure S3C and microscopy of sphere-formation assay in Figures 2C and 6B were redundantly used. The corrected Figures 2C, 6B, and S3C appear below.

**Figure 2:**
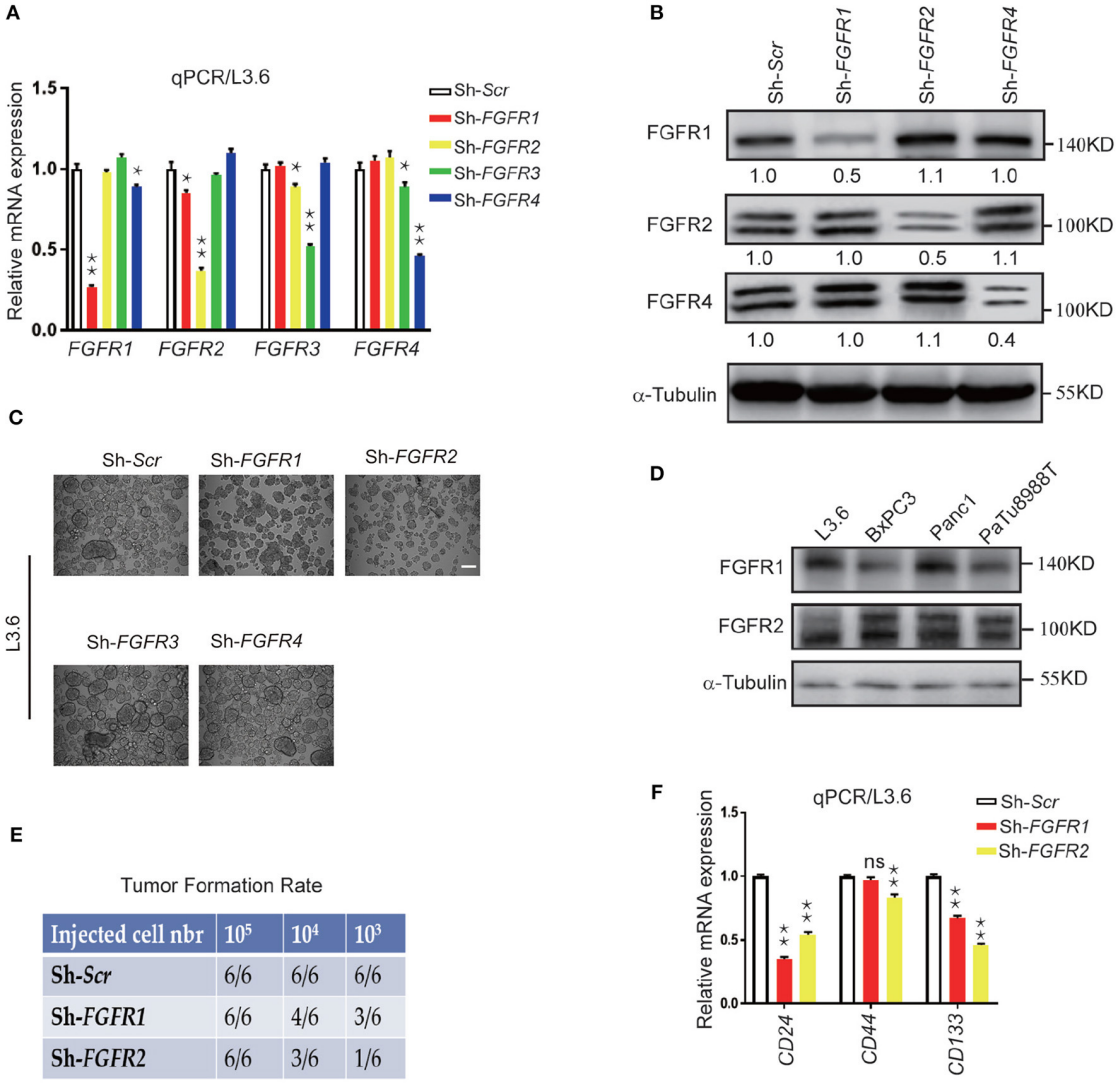
Genetic silencing of FGFR expression leads to reduced stemness *in vitro* and tumor formation *in vivo*. **(A,B)** Expression of *FGFRs* by qPCR and western blot in L3.6 cells upon silencing specific FGFRs. Numbers below the blots are quantifications for three independent experiments. **(C)** Sphere formation assay in L3.6 cells following specific FGFR knockdown and quantification of sphere numbers for three independent experiments. Scale bar: 200 μm. **(D)** FGFR1 and FGFR2 protein expression by western blot in several pancreatic cancer cell lines. **(E)** Tumor formation rate 3 weeks following the subcutaneous inoculation of different numbers of L3.6 cells to nude mice. **(F)** Expression of stemness markers *CD24, CD44*, and *CD133* by qPCR in L3.6 cells upon silencing of *FGFR1* or *FGFR2*. *p ≤ 0.05, **p ≤ 0.01.

**Figure 6:**
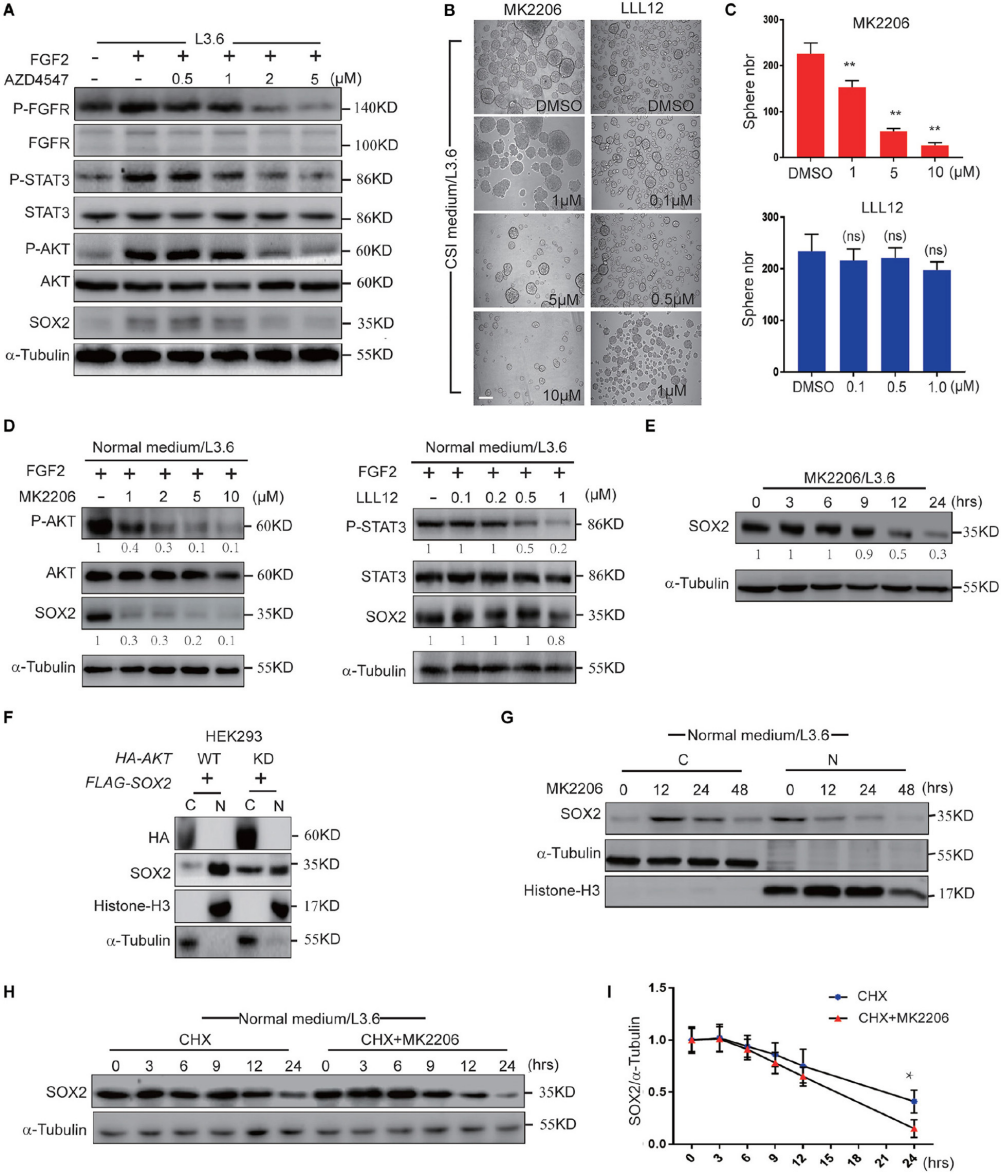
FGFR regulates SOX2 mainly through AKT. **(A)** Western blot analysis of key FGFR downstream pathways in L3.6 cells treated with different doses of AZD4547 together with FGF2 (10 ng/ml) for 12 h. **(B)** Sphere formation assay using L3.6 cells treated with different doses of MK2206 (AKT inhibitor) and LLL12 (STA3 inhibitor). Scale bar: 200 μm. **(C)** Corresponding sphere number quantification for three independent experiments. **(D)** Western blot analysis of pathway inhibition efficiency of MK2206 and LLL12 for 24 h. Numbers below the blots are quantifications for three independent experiments. **(E)** Western blot was performed to quantify SOX2 expression levels upon MK2206 (2 μM) treatment in L3.6 cells at indicated time points. Numbers below the blots are quantifications for three independent experiments. **(F)** SOX2 detection in cytoplasmic and nuclear fractions upon transfection with *AKT*-WT or *AKT*-KD in HEK293. **(G)** SOX2 detection in cytoplasmic and nuclear fractions upon MK2206 (2 μM) treatment at different time points. **(H)** Western blot was carried out to quantify SOX2 expression level upon treating with CHX (50 μg/ml) with and without MK2206 (2 μM) in L3.6 cells at different time points. **(I)** Corresponding quantification for three independent experiments. *p ≤ 0.05, **p ≤ 0.01.

**Figure S3:**
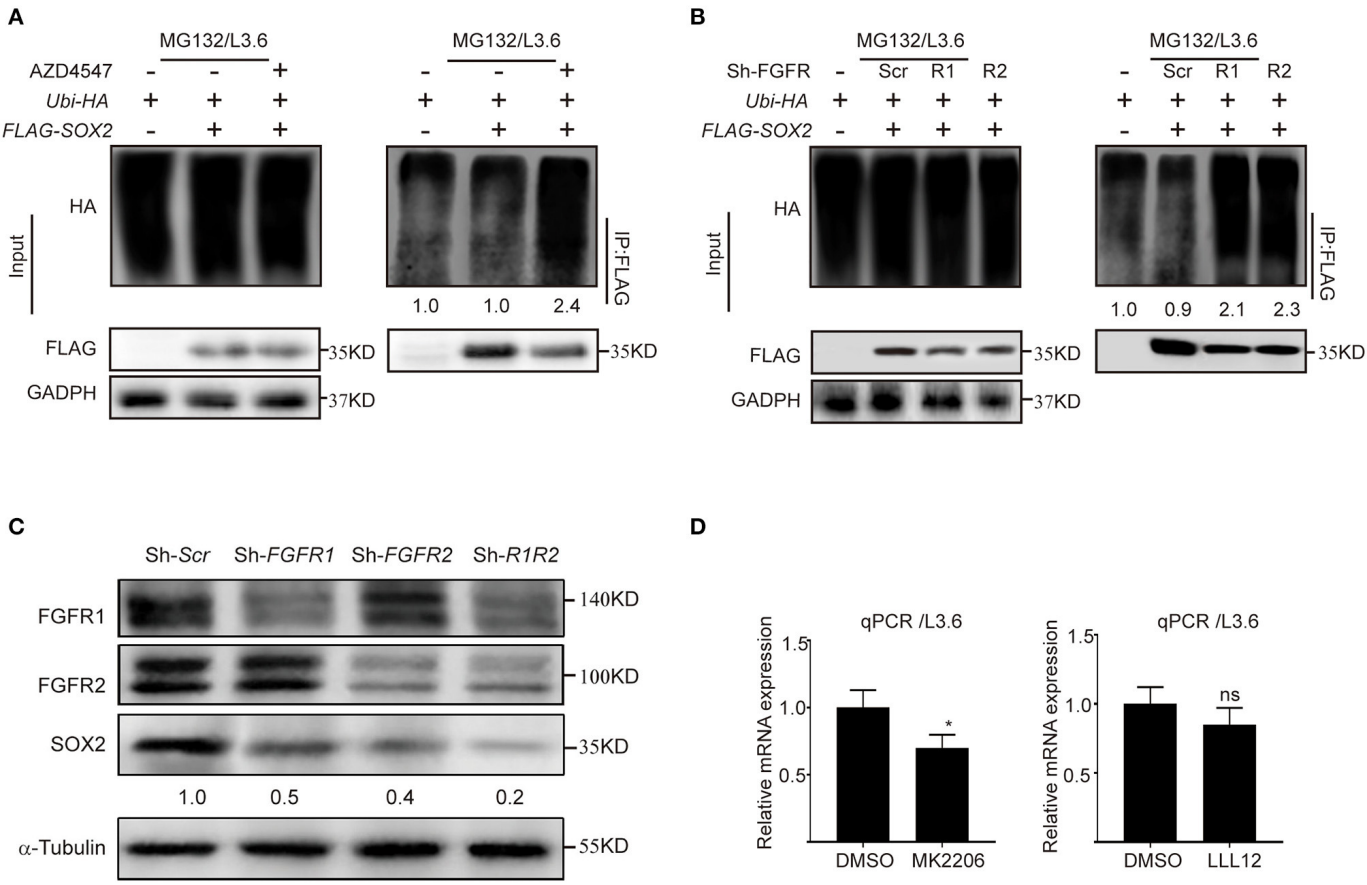
**(A)** Analysis of SOX2 ubiquitination in L3.6 cells with or without AZD4547 (2 μM) in the presence of MG132 (20 μM) to block degradation. Numbers below the blots are quantifications for the blots; **(B)** SOX2 ubiquitination analysis in control cells vs. FGFR1 and FGFR2 knockdown cells. Numbers below the blots are quantifications for the blots; **(C)** Western blot was used to detect FGFR1, FGFR2 and SOX2 expression upon *FGFR1, FGFR2* and double knockdown in L3.6 cells. Numbers below the blots are quantifications for the blots; **(D)** Quantification of *SOX2* mRNA levels following 24 h of MK2206 (1 μM) treatment or LLL12 (0.5 μM) treatment.

The authors apologize for these errors and state that these corrections do not change the scientific conclusions of the article in any way. The original article has been updated.

