# Supplementary material for: An FGFR/AKT/SOX2 Signaling Axis Controls Pancreatic Cancer Stemness
Source: Front Cell Dev Biol. 2020 May 7;8:287. doi: 10.3389/fcell.2020.00287 (PMC7221133; doi:10.3389/fcell.2020.00287)
Supplement: Supplementary file 1 [file Table_1.DOCX]

**Supplementary Information**

**Figure S1**

**
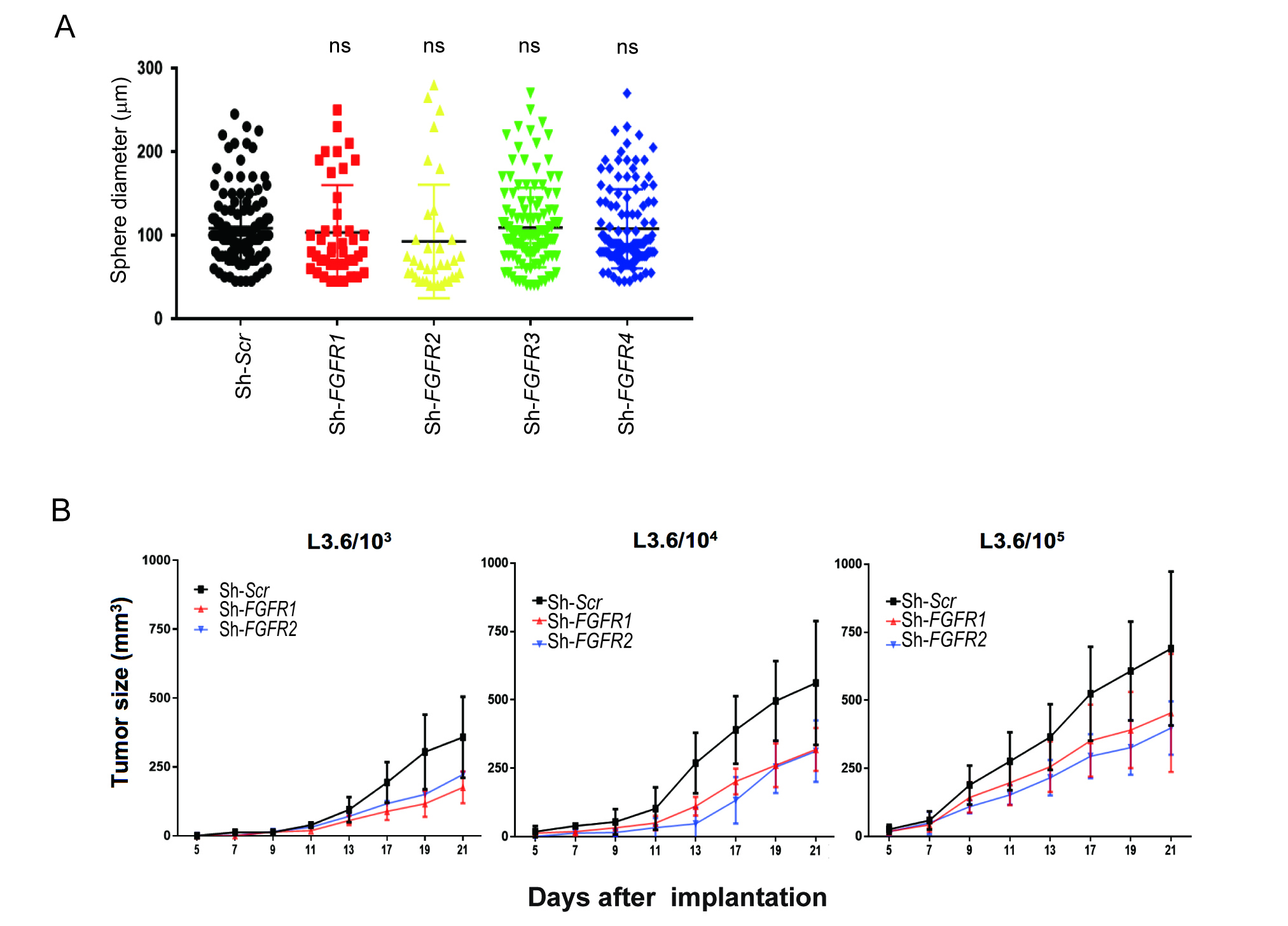
**

**Figure S1 Effect of FGFR knockdown on sphere formation *in vitro* and tumor growth *in vivo*** (A) Quantification of sphere diameter in scramble control vs. *ShFGFR* knockdown cell lines (each spot represents one sphere); (B) The kinetic curves of tumor growth for each group. Only occurred ones were recorded.

**Figure S2**

**
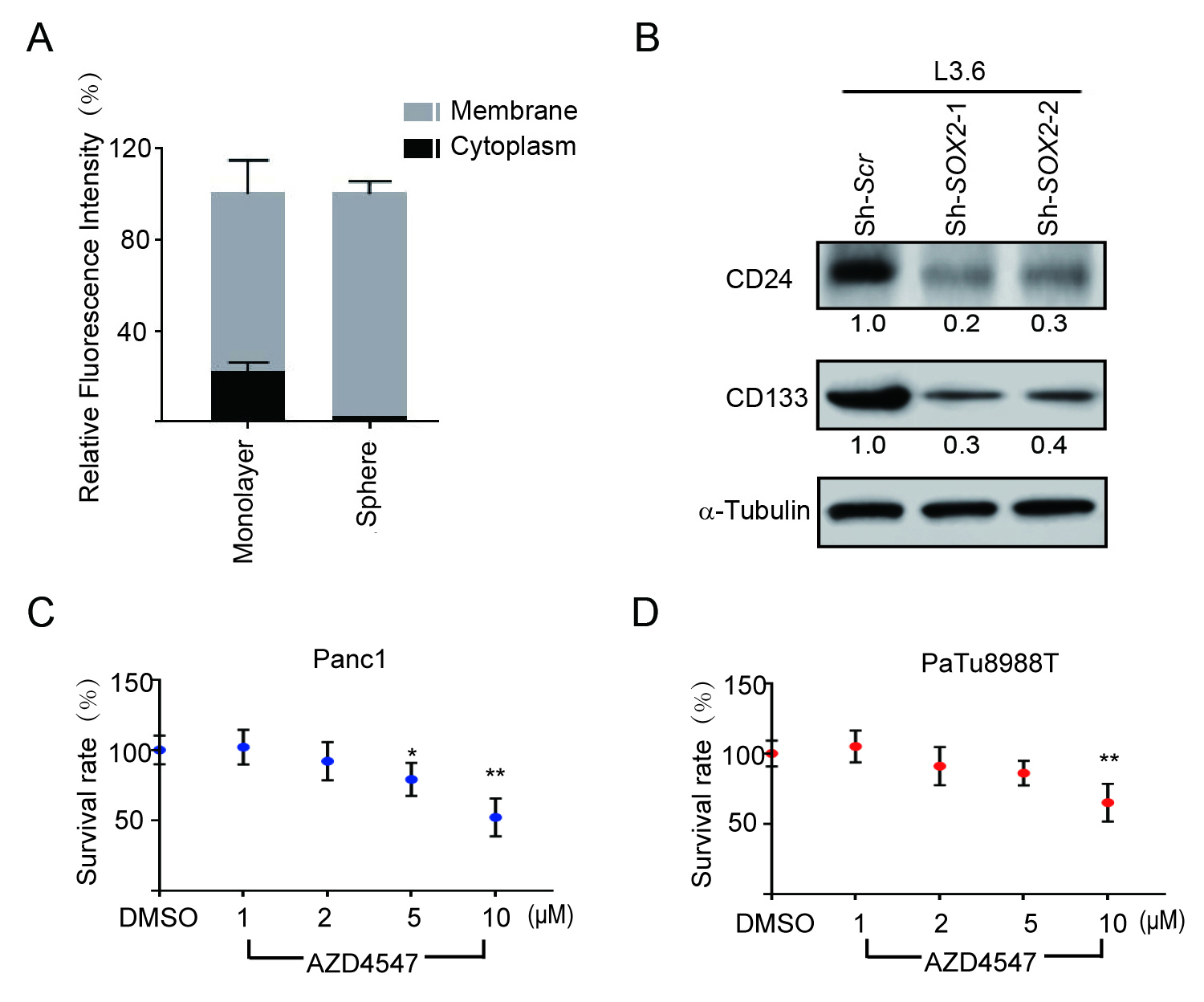
**

**Figure S2** (A) Quantification of CD24 distribution by immunofluorescence in Figure 3A; (B) Expression of stemness markers CD24 and CD133 by western blot in L3.6 cells upon silencing of *SOX2.* Numbers below the blots are quantifications for three independent experiments; (C, D) Cell survival rate in Panc1 and Patu8988T cells after 72 h treatment with indicated doses of MK2206 and LLL12 compared with DMSO, *p≤0.05, **p≤0.01.

**Figure S3**

**
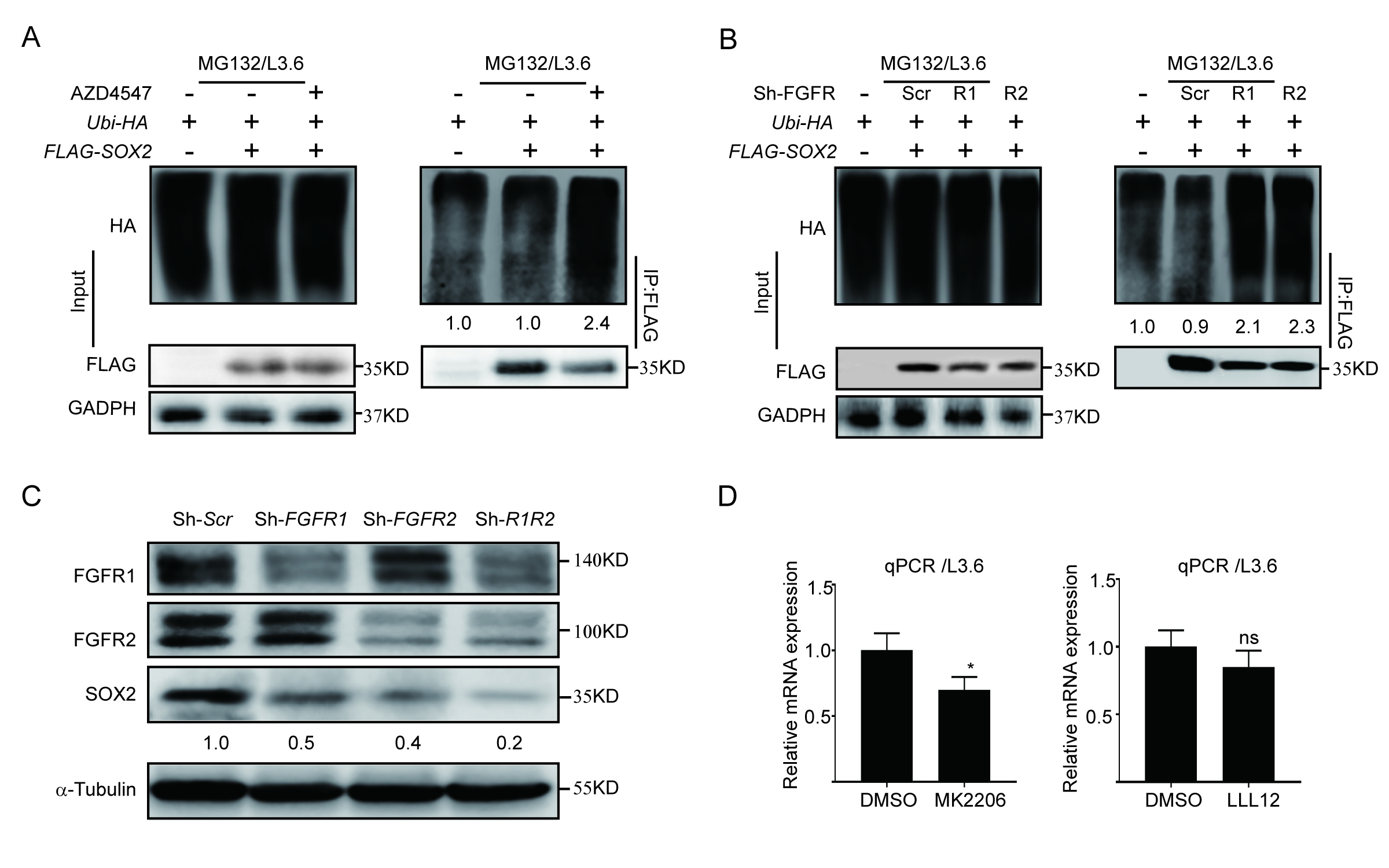
**

**Figure S3** (A) Analysis of SOX2 ubiquitination in L3.6 cells with or without AZD4547 (2μM) in the presence of MG132 (20μM) to block degradation. Numbers below the blots are quantifications for the blots; (B) SOX2 ubiquitination analysis in control cells vs. FGFR1 and FGFR2 knockdown cells. Numbers below the blots are quantifications for the blots; (C) Western blot was used to detect FGFR1, FGFR2 and SOX2 expression upon *FGFR1*, *FGFR2* and double knockdown in L3.6 cells. Numbers below the blots are quantifications for the blots; (D) Quantification of *SOX2* mRNA levels following 24 h of MK2206 (1μM) treatment or LLL12 (0.5μM) treatment.
