# Supplementary figures and images for: An FGFR/AKT/SOX2 Signaling Axis Controls Pancreatic Cancer Stemness
Source: Front Cell Dev Biol. 2020 May 7;8:287. doi: 10.3389/fcell.2020.00287 (PMC7221133; doi:10.3389/fcell.2020.00287)

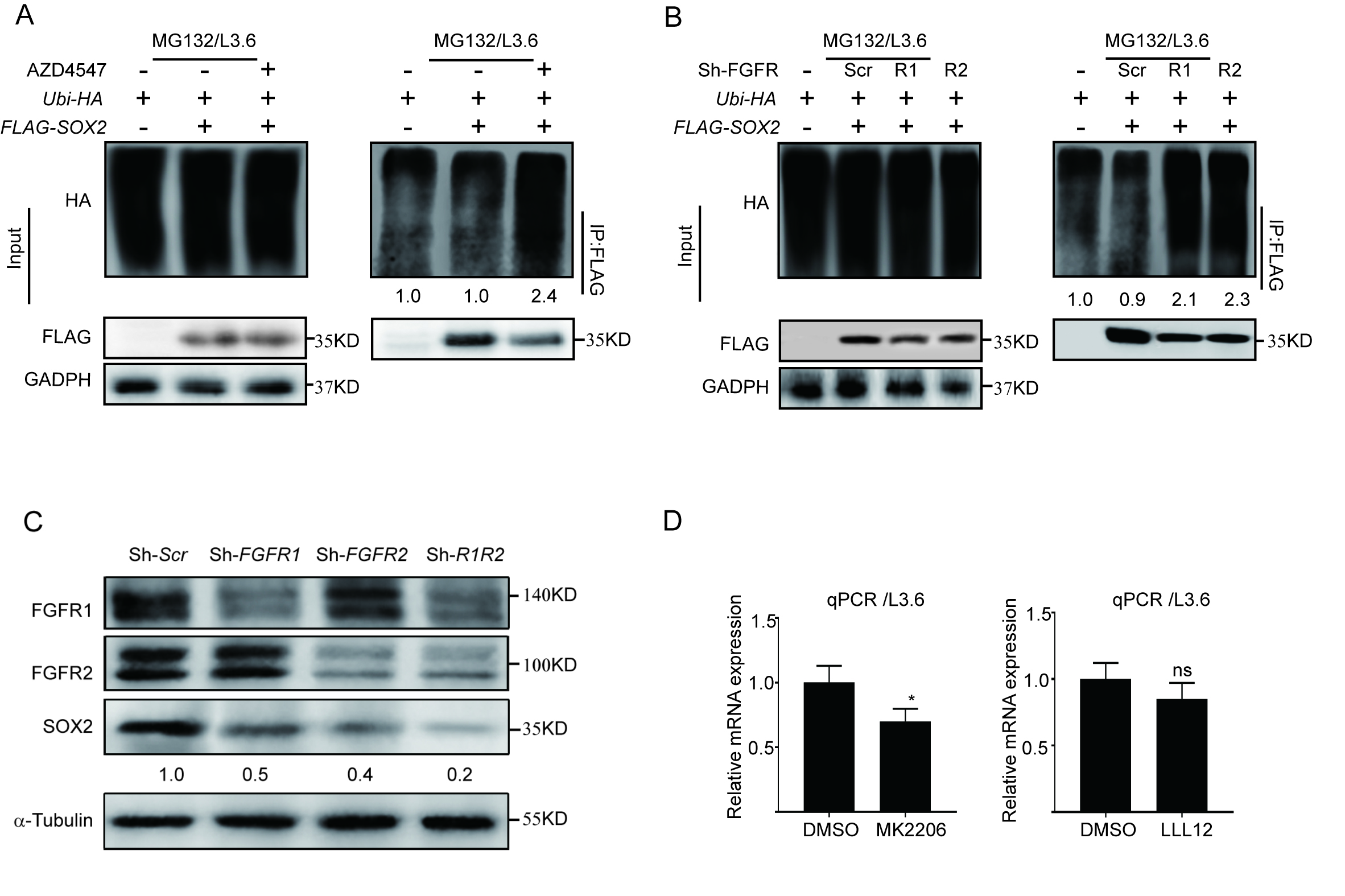

Supplement: Supplementary file 2 [file Image_3.jpg]
